# A Novel Approach of Groupwise fMRI-Guided Tractography Allowing to Characterize the Clinical Evolution of Alzheimer's Disease

**DOI:** 10.1371/journal.pone.0092026

**Published:** 2014-03-17

**Authors:** Maria Giulia Preti, Nikos Makris, George Papadimitriou, Maria Marcella Laganà, Ludovica Griffanti, Mario Clerici, Raffaello Nemni, Carl-Fredrik Westin, Giuseppe Baselli, Francesca Baglio

**Affiliations:** 1 IRCCS, Fondazione Don Carlo Gnocchi ONLUS, Milan, Italy; 2 Politecnico di Milano, Department of Electronics, Information, and Bioengineering, Milan, Italy; 3 Departments of Psychiatry and Neurology Services, Center for Neural Systems Investigations, Center for Morphometric Analysis, Athinoula A. Martinos Center for Biomedical Imaging, Massachusetts General Hospital, Harvard Medical School, Boston, Massachusetts, United States of America; 4 Department of Psychiatry, Psychiatry Neuroimaging Laboratory, Brigham and Women's Hospital, Harvard Medical School, Boston, Massachusetts, United States of America; 5 Department of Anatomy and Neurobiology, Boston University School of Medicine, Boston, Massachusetts, United States of America; 6 Department of Pathophysiology and Transplantation, University of Milan, Milan, Italy; 7 Department of Radiology, Laboratory of Mathematics in Imaging, Brigham and Women's Hospital and Harvard Medical School, Boston, Massachusetts, United States of America; Brainnetome Center, & National Laboratory of Pattern Recognition, China

## Abstract

Guiding diffusion tract-based anatomy by functional magnetic resonance imaging (fMRI), we aim to investigate the relationship between structural connectivity and functional activity in the human brain. To this purpose, we introduced a novel groupwise fMRI-guided tractographic approach, that was applied on a population ranging between prodromic and moderate stages of Alzheimer's disease (AD). The study comprised of 15 subjects affected by amnestic mild cognitive impairment (aMCI), 14 diagnosed with AD and 14 elderly healthy adults who were used as controls. By creating representative (*ensemble*) functionally guided tracts within each group of participants, our methodology highlighted the white matter fiber connections involved in verbal fluency functions for a specific population, and hypothesized on brain compensation mechanisms that potentially counteract or reduce cognitive impairment symptoms in prodromic AD. Our hope is that this fMRI-guided tractographic approach could have potential impact in various clinical studies, while investigating white/gray matter connectivity, in both health and disease.

## Introduction

The exploration of white matter (WM) microstructure and gray matter (GM) function is now widely available due to the great advancement of noninvasive techniques, such as diffusion tensor imaging (DTI) and functional magnetic resonance imaging (fMRI). DTI-based tractography is a virtual *in-vivo* reconstruction of WM fibers that represent anatomical connections among different brain regions [1]. fMRI is based on the premise that neuronal activation is directly linked to cerebral blood flow and oxygenation, thus it is used to detect functional brain activity by measuring the blood oxygenation level dependent (BOLD) signal [2], [3]. fMRI-guided tractography aims to integrate brain structure and function, by assessing which WM tracts have direct anatomic connection with a region of GM activity. It is based on a hypothesis that a WM tract directly involved in delivering relevant information (in the form of electrical impulses) to an active cortical area terminates within its very close proximity [4]. Given this assumption, fMRI-guided tractography allows to directly correlate WM structural connectivity with GM functional response, while assessing potential damage effects of neurodegenerative pathologies or brain lesions [5]. Functional loss of structural connections and alternate route possibilities due to plastic adaptation can be explored. We are looking to introduce a reliable tractographic methodology that will also allow structural WM connectivity comparisons among groups of population, thus extending the standard single-subject approach. Most studies currently available in the literature, in fact, focus their analyses on each subject's native acquisition space [6]–[10], with only a couple of exceptions [11], [12]. Bonzano et al. [11] used each participant's fMRI-driven tracts to build a probabilistic map of the connections for the study population. However, the volume-based probabilistic approach does not allow for the preservation of the 3D shape and trajectory of the fibers of interest. To our knowledge, no work in literature currently addresses the construction of an fMRI-guided 3D *ensemble* WM tract containing all fibers involved in a cortical activation, while preserving the 3D rendering shape and orientation of the fibers. Various clinical applications of fMRI-guided tractography have been explored, e.g. on multiple sclerosis [11], or brain tumors [6], and many more can potentially benefit in the field of neurodegenerative pathologies. For example, DTI tractography guided by cortical activated regions resulting from an fMRI task on semantic verbal fluency could be promising in the differentiation of healthy elderly subjects from moderate Alzheimer's Disease (AD) or amnestic mild cognitive impairment (aMCI), a condition widely considered of increased risk to develop AD [13]–[15].

Our work aims to apply groupwise fMRI-driven tractography to assess the semantic fluency activation patterns in normal aging, aMCI and AD. We hope to assess group differences in structural and functional connectivity, representing possible biomarkers of the AD clinical progression.

## Methods

### Ethics statement

The study was approved by the Don Gnocchi Foundation Ethics Committee (Milan, Italy) and was conformed to the ethical principles of the Helsinki Declaration. An informed written consent was obtained from all subjects.

### Subjects

Magnetic resonance (MR) images were acquired for 39 elderly healthy controls (HC) (14 subjects used as controls in the study: mean age 70.2±5.4, 6 males; 25 used for an atlas generation (aHC): mean age 70.2±5.1, 11 males), 15 subjects with aMCI (age 73.2±4.9, 8 males) and 14 patients affected by AD (age 75.6±5.4, 6 males), recruited at the Fondazione Don Gnocchi (Milan, Italy). aMCI subjects were diagnosed following Petersen criteria [13] and Grundman and colleagues operational criteria [16]: memory complaint, confirmed by an informant; abnormal memory function, documented by previous extensive neuropsychological evaluation; normal general cognitive function, as determined by both clinical dementia rating (CDR [17]) scale (CDR with at least 0.5 in the memory domain) and mini-mental state examination (MMSE [18]) score (MMSE greater than or equal to 24); no impairment in functional activities of daily living as determined by a clinical interview with the patient and informant; no significant cerebral vascular disease (Hachinski score less than or equal to 4 [19]); no major psychiatric illnesses with particular attention to exclude subjects with a history of depression (Hamilton depression rating scale score less than or equal to 12 [20]). Moreover, to increase the diagnostic accuracy of aMCI, we included aMCI subjects with at least one abnormal biomarker of neuronal injury, according to the guidelines for MCI due to Alzheimer's dementia [21]. Patients with AD were diagnosed according to the NINCDS-ADRDA criteria [22] and to the updated guidelines for AD of the National Institute on Aging Alzheimer's Association [23]; all AD patients were in mild to moderate stage of the disease according to CDR scale (0.5 to 2) and to MMSE score (between 18 and 23). HC were preliminarily screened to exclude major systemic, psychiatric or neurological illnesses.

### MR acquisition protocol

Brain MR acquisitions were performed using a 1.5 Tesla scanner (Siemens Magnetom Avanto, Erlangen, Germany), including the following sequences: 1) Dual-echo turbo spin echo (TR = 2650 ms, TE = 28/113 ms, echo train length = 5, flip angle = 150°, 50 interleaved 2.5-mm-thick axial slices, matrix size = 256×256 interpolated to 512×512, FOV = 250 mm×250 mm); patients' T2-weigthed scans were reviewed by an experienced neurologist to assure the absence of WM hyperintensities outside the normal range (no T2-weighted abnormalities located in the deep white matter and/or no more than five abnormalities located in periventricular regions, with diameter <5 mm). 2) Diffusion weighted (DW) pulsed-gradient spin-echo planar (TR = 7000 ms, TE = 94 ms, 50 2.5-mm-thick axial slices, matrix size  = 128×96, FOV = 320 mm×240 mm), with diffusion gradients (b-value  = 900 s/mm^2^) applied in 12 non-collinear directions. Two runs of images were acquired for each HC, with one b = 0 image without diffusion weighting for each run. The diffusion sequence parameters were tuned to optimize signal to noise ratio [24], allowing for the best desirable result compatible with time-limited clinical protocols and for a successful tract reconstruction, despite the limited available number of diffusion gradient directions. 3) Single-shot gradient echo EPI sequence (TR = 3000 ms, TE = 50 ms, matrix size  = 64×64, FOV = 250 mm×250 mm) using blood oxygenation level dependent (BOLD) contrast for functional imaging. Each session included 120 volumes consisting of 38 axial slices with a 3 mm thickness in order to cover the entire brain. 4) 3-dimesional T1-weighted magnetization prepared rapid gradient echo (MPRAGE) (TR = 1900 ms, TE = 3.37 ms, TI = 1.1 ms, flip angle  = 15°, 176 contiguous, axial slices with voxel size  = 1 mm×1 mm×1 mm, matrix size  = 192×256, FOV = 192 mm×256 mm, and slab thickness  = 176 mm) used as anatomical scan for fMRI analysis.

#### fMRI stimuli and design

In order to study the areas involved in language processing, we adopted the verbal fluency paradigm described by Basho and colleagues [25]. The paced overt version of this category-driven word generation protocol was chosen, because it allows for appropriate response monitoring and a tight control of individual variability in task performance, making it suitable for the application in patients with cognitive deficits. We used an MR-compatible visual system to present the stimuli (VisuaStim Digital system from Resonance Technology Inc.) and the use of E-Prime software (E-Prime 2.0 Psychology Software tool, http://www.pstnet.com) ensured exact timing of prompts during MR acquisitions. The performance in doing the task was assessed for all subjects (percentage of correctly uttered words).

### DTI analysis

For all subjects, DTI analysis and brute force reconstruction [26] of resulting tractographic fibers were performed. A standard reconstruction of main bundles based on anatomical landmarks (detailed here on) was performed only on the separate group of 25 aHC, leading to reference probabilistic tractographic atlases to be further used in the analysis of fMRI-guided tractography of HC, aMCI, and AD groups. For each HC, DW images (DWI) were corrected for eddy current distortions, and since two separate diffusion runs were acquired, all diffusion images were registered to the very first b = 0 (i.e. anatomical reference image: b-value is 0 s/mm^2^) of the first diffusion run. FMRIB software library (FSL, http://www.fmrib.ox.ac.uk/fsl/; functions eddy_correct and FLIRT) was used to estimate the transformation matrices. The diffusion tensor (DT) and the fractional anisotropy (FA) maps were estimated with Diffusion Toolkit (www.trackvis.org) v0.6, which firstly rotates the B-matrix for slice angulation and for the rotation applied. Tractography was performed with Diffusion Toolkit, using the brute force approach and the interpolated streamline deterministic algorithm. Conventional thresholds of angle  = 35° and FA = 0.2 were adopted as stopping criteria. The data from the 25 aHC acquired for atlas generation purposes were used to construct tractographic atlases of the following WM bundles: corpus callosum (CC), arcuate fasciculus (AF), inferior longitudinal fasciculus (ILF), inferior fronto-occipital fasciculus (IFOF), cingulum bundle (CB) and uncinate fasciculus (UF). The bundle tractographic reconstruction was performed with TrackVis (www.trackvis.org) by positioning regions of interest (ROIs) of waypoints on FA maps or FA color maps using the anatomical definitions of each bundle as described in [27]. The probabilistic approach introduced by Hua et al. [28] was adopted for the atlas construction. Then, the FA maps of the three study groups (HC, aMCI, AD) were nonlinearly registered on the atlas and atlas-based FA values were computed in all subjects as a measure of WM integrity for all the above-cited bundles, using the atlas-based tractography method detailed in [29]. The WM atlases of the elderly population are available upon request via e-mail.

### fMRI analysis

The fMRI data processing was carried out using FSL FEAT v5.98, with the default parameters suggested by FSL and reported here on. Pre-statistic processing included motion correction using FLIRT, non brain structures removal using BET, spatial smoothing with a Gaussian kernel of 5 mm and high pass filtering with a cutoff of 60 s. FILM was used to perform the time-series statistical analysis with local autocorrelation correction and motion parameters were included in the model as regressors. Z statistics images were thresholded using clusters determined by Z>2.3 and a corrected cluster significance threshold of p = 0.05. Four second-level analyses were carried out with FSL to assess the group average activations (an ANOVA test comparing the three groups and a one-sample t-test for each group). Registration to high-resolution structural images (T1-MPRAGE) was carried out using FLIRT. Registration from high resolution structural to standard space (Montreal Neurological Institute, MNI) template was then further refined using FNIRT nonlinear registration. The subjects' performance in doing the task was compared among the three groups of subjects with an ANOVA test with Bonferroni correction for multiple comparisons, and introduced as covariate in all fMRI analyses.

### fMRI-guided tractography: a novel approach for the group analysis

The proposed fMRI-guided tractographic approach is a groupwise fMRI and DTI intergration technique that was previously presented in a pilot study on a sample of HC [30]. The entire pipeline (as described in [30]) was here applied separately to three group populations (HC, aMCI, and AD), and resulted in three distinct fMRI-guided sets of *ensemble* tracts.

**Figure 1:**
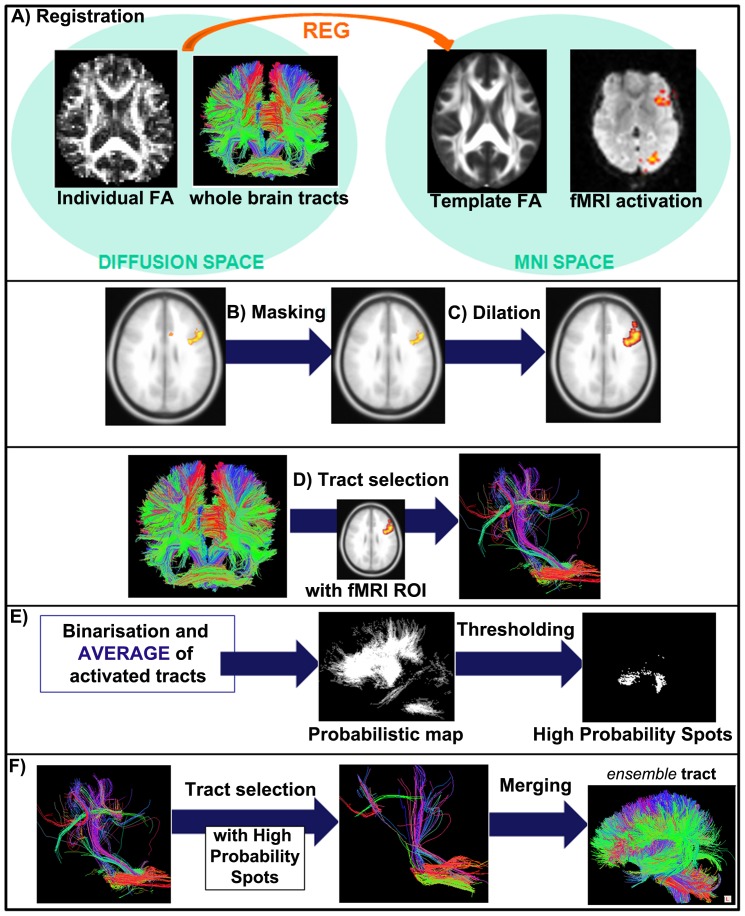
fMRI-guided tractography method: group analysis. Steps A–F described in the text (Materials and Methods section) are here graphically explained (A: Registration, B: Masking, C: Dilation, D: Individual fMRI-guided tractography, E: fMRI-guided probabilistic map, F: fMRI-guided *ensemble* tract). The final result is the *ensemble* tract, i.e. a tractographic template of the activated tracts in the considered population.

For every subject, preliminary steps (A–D) were performed to determine which WM tracts are contributing to a specific task-related functional GM activation (see Fig. 1). A) Registration. We registered the DTI tracts of each subject to the MNI template. The linear transformation between the FA map of each subject and the MNI template FA (FMRIB58_FA) was estimated using FLIRT and applied to each tract using TrackVis. No registration was required for the cortical activations, as the FSL-based second level fMRI (one sample t-test) output is already sampled on MNI template space. B) Masking. The fMRI activation regions resulting from the second level fMRI analysis (one sample t-test) were masked by a GM template in MNI space, to ensure the exclusion of erroneous voxels belonging to WM or cerebrospinal fluid (CSF). C) Dilation. A minimal 2 mm dilation (using a disk-shaped kernel) of the fMRI active regions was performed to obtain the waypoints ROIs extending into the outer layer of WM bordering the activated GM. D) Individual fMRI-guided tractography. All tracts with direct connections to the fMRI task-related waypoints ROIs were designated as “active”.

In an effort to extend our method to a group-level approach, we applied the following steps (E-F) to create fMRI-driven *ensemble* tracts for each population (HC, aMCI, and AD), that represent all “active” tracts associated with the verbal fluency task. Tract involvement is implicitly defined by a fiber's direct connection to the task-related active GM area. E) fMRI-guided probabilistic map. Binarized volume masks representing all “active” tract-intersected voxels (per subject) were generated using TrackVis. The resulting maps were averaged to obtain a group probabilistic map. F) fMRI-guided *ensemble* tract. The group probabilistic map was thresholded at a value of 0.7, thus selecting all voxels where an “active” tract is present for at least 70% of the group population. We chose not to consider probabilities <70% in order to select only the most represented areas in the group and use these to filter the individual tracts. All “active” tracts (of every subject) were in fact subsequently filtered through the thresholded probabilistic map and merged together to obtain a group representative “active” tractographic template. In this way, the main information about tract directions was restored, after a probabilistic selection. Further, the effect of arbitrary choosing a threshold on the probability map is diminished here (with respect to usual probability approaches), as in the final *ensemble* tract also voxels below the chosen threshold are included, if belonging to a selected (“active”) fiber (i.e. a tract passing through high probability regions).

### Quantitative evaluation of different WM bundle involvement

The resulting fMRI-guided *ensemble* tract volumes for the three groups were superimposed on the anatomy-based tractographic atlases constructed on the aHC population, in order to obtain a groupwise quantitative measure of task involvement for each WM bundle. Tract density maps of the fMRI-guided *ensemble* tracts were overlayed on the WM atlases. For each tract separately, its percentage of involvement was computed as the number of “active” voxels (common voxels between aHC WM atlas and fMRI-guided *ensemble* tract), divided by the total number of voxels of the aHC WM atlas.

## Results

The demographic data of the study population is shown in Table 1. No significant differences were found in age and gender among all groups.

**Table 1:** Demographic data of the study groups.

	HC (N = 14)	aMCI (N = 15)	AD (N = 14)
**Age (years) (Mean [SD])**	70.2 [5.4]	73.2 [4.9]	75.6 [5.4]
**M/F**	6/8	8/7	6/8
**CDR (Median [range])**	0 [0]	0.5 [0–0.5]	1 [0.5–1]
**MMSE (Mean [SD])**	28.45 [1.76]*	25.93 [1.80]*	21.25 [1.78]*

Chi square was used for gender comparison. One-way ANOVA test with Bonferroni correction for multiple comparisons was used for age and MMSE score comparisons (significance level: pcorr<0.05).

Abbreviations: HC  =  healthy controls; aMCI  =  amnestic mild cognitive impairment; AD =  Alzheimer's disease; M/F =  males/females; CDR =  clinical dementia rating scale; MMSE =  mini-mental state examination; SD =  standard deviation.

*All differences resulted significant between the groups.

The average performance in the verbal fluency task was lower in the AD population (mean ± SD  = 86, 58±7,28), and progressively increased in the aMCI (mean ± SD  = 92,86±7.12) and HC group (mean ± SD  = 98.81±2.32), respectively. All differences between groups were found to be significant (ANOVA with Bonferroni correction for multiple comparisons, p<0.05).

**Figure 2:**
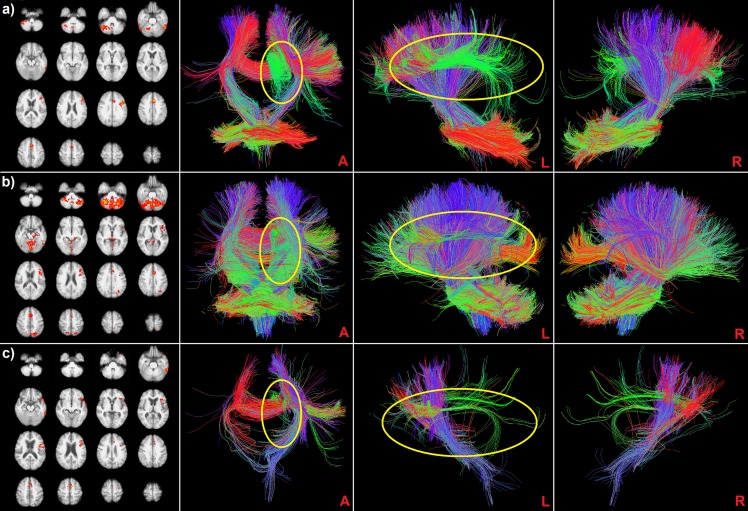
fMRI activation maps and fMRI-guided *ensemble* tracts for a) HC, b) aMCI and c) AD. From left to right, PANEL 1: second level fMRI group activation map resulting from one sample t-tests performed separately for each group, verbal fluency task, axial views; PANELS 2/4: anterior (A), left lateral (L) and right lateral (R) views of the *ensemble* tract based on the activation of the first panel. The yellow circles highlight the left CB (anterior view) and the left AF (left view), observable with different portions in all the three groups.

The one sample t-tests performed for each group showed different patterns of significant fMRI activations (cluster coordinates are reported in Table 2). For the HC, the activation was located in the left hemisphere, focusing on the inferior frontal gyrus, anterior cingulate cortex, superior frontal and fusiform gyrus. Together, a modest activation regarding the controlateral cerebellum (right hemisphere) was observable (Tab. 2, Fig. 2, a). The same pattern of activation detected for the control group was found for the aMCI, but with a larger involvement of the cerebellum (bilateral) and an additional activation of the bilateral parietal cortex (Tab. 2, Fig. 2, b). Instead, the average activation of the AD group was considerably weaker, but nonetheless following the activation pattern of the controls (Tab. 2, Fig. 2, c). For AD patients, however, no significant activation was found in the cerebellum. Differences in cerebellum activations were confirmed by the direct comparison among the three groups performed with the ANOVA test (MCI>AD: peak value Z = 4.52, x = 30, y = −50, z = 23, with k = 42763; MCI>HC: peak value Z = 3.58, x = −19, y = −78, z = −31, with k = 4681).

**Table 2:** The main effect of verbal fluency task in HC (n = 14), subjects with aMCI (n = 15) and AD (n = 14), who completed the fMRI assessments.

Group	Cluster size	X	Y	Z	Side	Brain Area	BA	Z-value
**HC**	4049	−50	16	30	L	Frontal_Inf_Gy	44	3.93
	4782	−7	18	41	L	Cingulum_Mid	32	4.54
		−3	19	40	L	Frontal_Sup_Medial/SMA	6	4.53
	12352	41	−58	−36	R	Cerebellum_Crus1		3.81
	3221	−54	−47	−23	L	Fusiform_Gy/Temporal_Inf	37/20	4.24
**aMCI**	6797	−44	12	17	L	Frontal_Inf_Gy	44	4.16
	4907	−4	17	41	L	Cingulum_Mid	32	3.98
		−5	15	42	L	Frontal_Sup_Medial/SMA	6	3.94
	72888	37	−56	−34	R/L	Cerebellum_Crus1		5.57
	7850	−5	−75	50	L	Precuneus/Parietal_Sup	7	3.88
		16	−64	52	R	Precuneus/Parietal_Sup	7	3.61
**AD**	10095	−45	11	21	L	Frontal_Inf_Gy	44/47	4.61
	3880	−7	24	31	L	Cingulum_Mid	32	3.82
		−1	4	54	L	Sup_med_Frontal_Gy/SMA	6	4.16
	4308	−55	−42	−23	L	Fusiform_Gy/Temporal_Inf	20/37	3.7

One sample t-test with cluster correction (Z>2.3), significance threshold of p = 0.05. X, Y, Z coordinates expressed in millimiters.

Abbreviations: Sup  =  superior; Inf  =  inferior; Mid  =  middle; R  =  right; L  =  left; Gy  =  gyrus; SMA  =  supplementary motor area; BA  =  Brodmann area; HC  =  healthy controls; aMCI  =  amnestic mild cognitive impairment; AD  =  Alzheimer's disease.

In Fig. 2, the resulting fMRI-guided ensemble tracts for the three groups are shown. The tract involvement reflected the fMRI activation pattern, so the *ensemble* tracts for the aMCI population appeared larger than in the moderate AD group. A common pattern of involvement regarding the left CB and AF was found in all of the three groups, although different portions of these bundles were included. Even at a visual inspection, one can notice that HC tracts were involved in the task to a much larger degree than in the patient groups. A precise measure of the WM bundle recruitment is provided in Table 3, containing the percentages of activation of the different WM bundles. The bundles that resulted to be involved with a higher percentage during the semantic verbal fluency task were the left AF and the CB, with higher percentages for the HC (AF = 75% and CB = 45% respectively), followed by aMCI (AF = 61% and CB = 25%) and AD, with really low percentages of involvement (AF = 21% and CB = 15%). Interestingly, the right CB was activated with a higher percentage for the aMCI (48%), with respect to the HC (33%) and AD group (11%), as well as the parietal and temporal CC.

**Table 3:** Measure of integrity and percentages of involvement in the activation of WM bundles for the three groups.

WM Bundle	HC		aMCI		AD	
	Mean FA [SD]	%	Mean FA [SD]	%	Mean FA [SD]	%
**Left CB**	0.39 [0.02] ^(*)^	75%	0.38 [0.02] ^(#)^	61%	0.36 [0.02] ^(*) (#)^	21%
**Left AF**	0.39 [0.01]	45%	0.38 [0.02]	25%	0.37 [0.02]	15%
**Right CB**	0.41 [0.02] ^(*)^	33%	0.40 [0.03] ^(#)^	48%	0.38 [0.02] ^(*)(#)^	11%
**Sup. par. CC**	0.47 [0.03] ^(*)^	24%	0.46 [0.03]	36%	0.44 [0.04] ^(*)^	5%
**Post. par. CC**	0.52 [0.03] ^(*)^	32%	0.51 [0.04]	32%	0.48 [0.04] ^(*)^	10%
**Temp. CC**	0.58 [0.02] ^(*)^	17%	0.57 [0.04]	29%	0.55 [0.04] ^(*)^	7%
**Left temp. CC**	0.62 [0.05] ^(*)^	12%	0.58 [0.08]	18%	0.57 [0.08] ^(*)^	9%
**Right temp. CC**	0.56 [0.03] ^(*)^	4%	0.54 [0.06]	12%	0.51 [0.08] ^(*)^	3%

The percentages over 5% in at least one group are reported. For each group, the percentages of involvement were computed superimposing the *ensemble* tract to the atlases of the WM fiber bundles and represent the number of overlapping voxels between the two, with respect to the number of voxels of the atlas. Abbreviations: WM  =  white matter; HC  =  healthy controls; aMCI  =  amnestic mild cognitive impairment; AD  =  Alzheimer's disease; FA  =  fractional anisotropy; SD  =  standard deviation; CB  =  cingulum bundle; AF  =  arcuate fasciculus; CC  =  corpus callosum; sup  =  superior; par  =  parietal; post  =  posterior; temp  =  temporal.

(*)The average FA was found to be significantly different between AD and HC (p<0.05 corrected for multiple comparisons).

(#)The average FA was found to be significantly different between aMCI and AD (p<0.05 corrected for multiple comparisons).

Average FA values found in the activated bundles for the three groups are also shown in Table 3, highlighting a diffuse WM damage regarding the AD condition (bilateral CB, parietal and temporal CC FA resulted significantly lower with respect to HC, p<0.05 corr.) and a general preservation of WM integrity in aMCI, especially regarding bilateral CB (both CB FA resulted significantly higher than AD group, p<0.05 corr., while the remaining bundles showed FA values in-between the HC and AD groups, not significantly different from them).

## Discussion

The present study aimed at introducing a robust groupwise method of combination between fMRI and tractography, in which a set of *ensemble* tracts is generated for a specific group population. An *ensemble* tract is a 3D model representation of the WM tractographic trajectories of “active” fibers that directly connect to GM areas showing activity during an fMRI task. The *ensemble* tract generation provides a standardized approach capable of groupwise examinations, facilitating inferences on a common set of pattern similarities or differences among populations. This can be invaluable in clinical studies, where one can choose to focus on general cognitive brain circuitry mechanisms, neural dysfunctions (e.g. structural loss of connectivity) or natural cognitive compensation mechanisms (e.g. alternate connectivity routing through neural plasticity), but that always require a groupwise analysis. The group-level aspect of the *ensemble* tract approach that we introduced is that we chose to focus on comparisons on a base of common within-group WM connectivity patterns among different populations, rather than getting hindered by each individual subject's highly variable structural connective morphology. The identification of the active cortical GM areas during the functional task was also done using a groupwise approach. In fact, instead of filtering each subject's tracts by its own fMRI activation maps, a one-sample t-test was used within each group to create a common set of GM activations per group (see Materials and Methods section). Therefore, in contrast to past literature on fMRI-guided tractography that primarily focused on single-subject analysis [6]–[10], our study extended to a group-level methodology, that has only been partially investigated [11], [12]. On one hand, a subject-specific analysis has the clear advantage of evaluating with higher precision the morphology of a specific individual, and this can be useful in different contexts, i.e. surgical planning. Here, on the other hand, we wish to detect the common features of a group, highlighting abnormalities that characterize a specific pathology and allowing for group comparisons between different populations. The innovation in our groupwise approach is the enhancement of a standard volume-based probabilistic map [11], by the generation of *ensemble* tracts, which maintain the WM connective shape and morphology. Voxelwise probabilistic approach primarily focuses on voxels of high inter-subject overlap (set by a threshold), but instead the goal of this work was to qualitatively compare (among different groups) entire tracts passing through these high probability voxels of interest. For this purpose, we chose to instead use a probabilistic map as a way to filter the fMRI-guided tracts, and specifically follow them along their path, part of which might be in areas where inter-subject overlap falls below the set threshold. This can be particularly relevant when dealing with a damaged neural substrate, subjected to higher inter-subject variability, as it happens in neurodegenerative pathologies [31], [32]. We tested our approach in the field of neurodegenerative diseases, such as AD, where cortical atrophy has previously been associated with structural connectivity deterioration [31]. We showed differences in GM and WM regional activity among three group populations (normal aging, prodromal and mild moderate stage of AD) during a paced-overt verbal fluency task. It is important to clarify that the size of a groupwise WM *ensemble* tract does not represent the bundle size, but rather the degree of involvement in the functional task. A more complete characterization of a group population (HC, aMCI or AD) should also include information about WM integrity, thus we also examined diffusion properties (e.g. mean FA) across the bundles of interest. The pattern of WM degradation that was observed was consistent with the typical evolution of AD pathology. In the moderate AD group, we noted an initial involvement of the CB and the middle posterior region of the CC (Tab. 3) [33], while a general preservation of integrity in aMCI was observed and could be an indicator that this is a transition phase between HC and AD (all FA values ranged in-between the two groups). This pattern of structural damage appears consistent with the results from the fMRI-guided tractography analysis. In normal aging, we observed a predominant involvement of the left cingulum bundle and the left arcuate fasciculus (Tab. 3), coupled with fMRI activations of the inferior frontal gyrus and the anterior cingulate cortex (Tab. 2). We noted reduced tract involvement for left CB and AF in the aMCI population, while there was an activation increase for other WM bundles (right cingulum bundle and parietal/occipital CC portions, see Tab. 3). This WM engagement regarding the aMCI subjects reflects both the GM pattern of activation (additional GM activations of bilateral parietal cortex and cerebellum and the functional loss in left temporal areas) and the pattern of WM integrity (preservation of bilateral CB with respect to the AD group) (see Tab. 2). The recruitment of the lateral portions of the cerebellum is consistent with recent studies showing its involvement in a broad spectrum of linguistic functions, including verbal fluency [34]–[37]. Specifically, the cerebellum participates in language networks with the activation of different cerebro-cerebellar circuits including the connections with left inferior frontal gyrus and left lateral temporal cortex [38]. Finally, the *ensemble* tract of the AD group appears to be a hypo-representation of the HC pattern (Fig. 2). This result primarily reflects diminished brain functioning in AD patients, as confirmed by their low performance score during the functional task, and the observed WM structural integrity deterioration, as demonstrated by the reduction in mean FA. We can argue that these findings indicate the usage of alternate brain GM/WM resources (engagement of bilateral CB, posterior portions of CC, and the bilateral activation of the cerebellum) only in aMCI to account for the lack of activation in lateral temporal cortex and the deterioration of WM structural integrity. We could hypothesize that these patterns represent a compensatory process reflecting a degree of neural plasticity in the aMCI group, as an attempt to preserve adequate brain function. This appears no longer possible for AD patients, possibly due to serious structural damage and connective degeneration, also reflected by poor functional task performance.

Since this was the first clinical validation of the proposed *ensemble* tract approach, our post-analysis included a comparison with an independently constructed anatomical tractographic atlas, which was entirely based on an elderly healthy control population. Tract involvement percentages were extracted by calculating the overlap between our group *ensemble* tracts and our anatomical atlas. The *ensemble* fMRI-driven tracts are open to a wealth of potential future analyses, since they contain information on the entire tract connecting different active cortical areas, rather than just WM voxels of high inter-subject overlap. For instance, active fMRI regions could be thought of as nodes and the *ensemble* tract fibers as weighted connections of a task-related structural and functional system network. Further, the future perspective of using this group-specific DTI-fMRI characterization for multimodal classification techniques appears indeed interesting and could represent a powerful additional means in AD/MCI diagnosis [39].

A possible limitation of this method that should be noted is the setting of certain parameters that are still currently based on empirical observations, and not yet grounded on a more theoretical basis. One such assumption is that WM neighboring a GM active cortical area contains axons that are involved in the specific activation. Anyway, still little is known about the WM organization and axons pathways when descending from columns in gray matter into white matter. They could in fact remain together or contrarily intermingle with fibers from more distant cortical regions [4]. A more direct validation that could yield greater understanding of the WM organization and its association with DTI tractography could come from comparing DTI data to neurotracer injections, not feasible in humans due to their invasive nature [4]. Further, future studies specifically investigating on the fibers connecting two activated GM areas would be possibly useful for a further understanding of the still unclear tract engagement mechanisms in the activation. However, the group analysis that we performed helped in showing the reliability of the results and highlighting the capability of the method in evaluating changes in anatomical and functional connectivity among different groups of patients.

In conclusion, we introduced a novel method of group-level fMRI-guided tractography that allows for the construction of fMRI-guided *ensemble* tracts representing WM fibers underlying specific functional cortical activity. Integrating diffusion tensor imaging as a representation of microstructural integrity and fMRI as an indication of brain functional activity can provide insight into the pathogenesis of neurodegenerative diseases. It further offers the opportunity to investigate *in vivo* the pathophysiological changes across the clinical evolution of AD pathology. These findings could potentially play a role in the diagnosis and prognostic assessment of early cognitive deficits in AD patients, and in subsequent longitudinal prospective studies.
